# Prevalence of sexually transmitted infections among pregnant women with known HIV status in northern Tanzania

**DOI:** 10.1186/1742-4755-6-4

**Published:** 2009-02-25

**Authors:** Sia E Msuya, Jacqueline Uriyo, Akhtar Hussain, Elizabeth M Mbizvo, Stig Jeansson, Noel E Sam, Babill Stray-Pedersen

**Affiliations:** 1 Department of International Health, Institute of General Practice and Community Medicine, University of Oslo, Oslo, Norway; 2 Department of Obstetric and Gynecology, Rikshospitalet University Hospital, University of Oslo, Oslo, Norway; 3 Department of Microbiology, Ullevaal University Hospital, University of Oslo, Oslo, Norway; 4 Kilimanjaro Christian Medical Centre, Moshi, Tanzania; 5 Ministry of Health and Child Welfare, Harare, Zimbabwe

## Abstract

**Objectives:**

To determine the prevalence of sexually transmitted infections (STIs) and other reproductive tract infections (RTIs) among pregnant women in Moshi, Tanzania and to compare the occurrence of STIs/RTIs among human immunodeficiency virus (HIV)-infected and uninfected women.

**Methods:**

Pregnant women in their 3^rd ^trimester (N = 2654) were recruited from two primary health care clinics between June 2002 and March 2004. They were interviewed, examined and genital and blood samples were collected for diagnosis of STIs/RTIs and HIV.

**Results:**

The prevalence of HIV, active syphilis and herpes simplex virus – type 2 (HSV-2) were 6.9%, 0.9% and 33.6%, respectively, while 0.5% were positive for *N gonorrhoeae*, 5.0% for *T vaginalis *and 20.9% for bacterial vaginosis. Genital tract infections were more prevalent in HIV-seropositive than seronegative women, statistically significant for syphilis (3.3% vs 0.7%), HSV-2 (43.2% vs 32.0%), genital ulcers (4.4% vs 1.4%) and bacterial vaginosis (37.2% vs 19.6%). In comparison with published data, a declining trend for curable STIs/RTIs (syphilis, trichomoniasis and bacterial vaginosis) was noted.

**Conclusion:**

Rates of STIs and RTIs are still high among pregnant women in Moshi. Where resources allow, routine screening and treatment of STIs/RTIs in the antenatal care setting should be offered. Higher STIs/RTIs in HIV-seropositive women supports the expansion of HIV-counseling and testing services to all centers offering antenatal care. After identification, STIs/RTIs need to be aggressively addressed in HIV-seropositive women, both at antenatal and antiretroviral therapy care clinics.

## Background

Sexually transmitted infections (STIs) are a major public health problem, especially in developing countries [1,2]. They are highly prevalent among pregnant women in Africa and cause significant maternal and perinatal morbidity [2-6]. STIs and other reproductive tract infections (RTIs) have been associated with a number of adverse pregnancy outcomes including abortion, stillbirth, preterm delivery, low birth weight, postpartum sepsis, neonatal pneumonia, neonatal blindness & congenital infection [1-4]. In addition, STIs/RTIs have been shown to facilitate transmission of HIV [7-12]. The control of STIs/RTIs, especially in pregnancy, is thus a priority, particularly in resource-poor settings where they are prevalent.

The prevalence of STIs however, has been shown to vary from one country to another and among different groups within the same country [2]. In Tanzania, the difference of STI/RTI prevalence by region, population and time-period has been observed [3,6,13-18]. There is thus a need to have local knowledge of the epidemiology of STIs/RTIs by periodically monitoring the prevalence of etiological agents. This information is useful in guiding clinical management, treatment protocols and to form the basis for STI surveillance. In this paper we report the prevalence of STIs/RTIs among pregnant women attending routine antenatal care in Moshi urban, Tanzania, as well as comparing the occurrence of infections among HIV-seropositive and sero-negative women. In addition, we compared the results of this study with previous published data on the prevalence of sexually transmitted infections among pregnant women in the same clinics.

To simplify the reading of the text, the acronym RTIs is used exclusively and is to be understood to include STIs as well as other non sexually transmitted diseases of the reproductive tract (i.e. bacterial vaginosis and candidiasis).

## Methods

### Study sites and population

The study was conducted among pregnant women attending routine antenatal care at two of the largest primary health care clinics (PHC) (Majengo and Pasua) in Moshi urban district. The district is the capital of Kilimanjaro region, situated in Northern Tanzania with the population of about 230,000 people [19]. The two clinics are the largest PHC and also represent women from the largest geographical areas (administrative wards). Attendance for antenatal care in this district is very high (> 97%) [19].

Eligibility criteria and recruitment procedures have been described in detail elsewhere [20]. Briefly, from June 2002 to March 2004, a cohort of 2654 pregnant women, in their third trimester, were invited to participate in a larger project which aimed to describe the acceptability of HIV/RTI perinatal interventions, especially prevention of mother-to-child transmission of HIV at the PHC level as well as to determine factors associated with incident HIV and RTIs in the postpartum period. This was preceded by a pilot project in 1999 which aimed primarily at determining the prevalence of RTIs among attendees, both pregnant and non-pregnant, at primary health care clinics in the same centres [18].

After obtaining informed consent, the participants underwent counseling followed by a structured interview in Swahili to collect information on socio-demographic variables, sexual behavior, obstetric history, and on current and past RTI symptoms. After the interview, a general and gynecological examination was performed. Genital ulcers, warts and abnormal vaginal or cervical discharge were diagnosed clinically. Vaginal and cervical samples were collected for diagnosis of various reproductive tract and sexually transmitted infections. Venous blood was taken for serological analysis of HIV, syphilis, HSV-2 and *Chlamydia trachomatis*. Genital samples were not collected from 99 of the women (4%) because they did not want to undergo speculum examination. The same procedures as above were followed in the pilot study in 1999.

Pregnant women with symptoms or signs suggestive of RTIs were treated on the spot following the Tanzanian Syndromic STI Case Management Guidelines. The women were asked to return for their HIV/RTI results after one week. Women found later, on laboratory testing, to have untreated infections were treated for specific infections during the follow-up visit. HIV positive women were given a single dose of Nevirapine for prevention of mother-to-child transmission (PMTCT) of HIV at post test counseling and their infants were given Nevirapine syrup within 72 hours after delivery. All of the women were encouraged to inform their partners and to bring them for counseling and testing. Those with proven RTIs were given a contact card to give to their partners so that they could come for treatment. All the services were free of charge for both the women and their partners.

Approval for the study was obtained from the Tanzanian Ministry of Health and The Regional Committee for Medical Research Ethics; Region III (Regional komite for medisinsk forskninsetikk region III)

### Laboratory methods

Active syphilis was diagnosed by positive results of both the rapid plasma regain test (RPR; Becton Dickinson, MD, USA) and a specific test, Determine Syphilis TP (Abbott Laboratories, IL, USA). HSV-2 was detected by the type-specific HSV-2 ELISA test (Focus Diagnostics, Cypress, California USA). HIV was diagnosed by a positive result on both the Determine HIV 1/2 (Abbott Laboratories, IL, USA) and Capillus HIV 1/2 (Trinity Biotech, Ireland). Discordant results (< 1%) were resolved by an ELISA test, Vironostika HIV Uni-form II (Organon Teknika, Boxtel, Netherlands). IgG antibodies to *Chlamydia trachomatis *was assessed in serum by an ELISA test (Ani Labsystems Ltd, Finland).

Trichomoniasis was diagnosed using wet preparation, candidiasis by visual inspection of *Candida *species on potassium hydroxide (KOH) preparations or on Gram-stained vaginal swabs. Bacterial vaginosis was diagnosed on the basis of Amsel's criteria [20]. N *gonorrhoeae *was diagnosed by culture (modified Thayer-Martin medium) and/or positive Gram-stained of endocervical swabs for Gram-negative diplococci.

### Statistical analysis

The data were entered and analyzed using SPSS statistical software, version 12.0 (SPSS, Chicago, IL, USA). Descriptive statistics were obtained through frequencies and cross tabulations. Comparison between groups was made using the χ^2 ^tests and Fisher exact test when appropriate. All analyses were two-tailed and the level of significance was set at 5%.

## Results

The age of the 2654 participating women ranged from 14–43 years (median 24 years), parity from 0–9 (median 1) and gestation age from 28–40 weeks (median 30 weeks). Other demographic details are described in Table 1.

**Table 1:** Socio-demographic, behavioural and obstetric characteristics of the 2654 participating women in Moshi urban district, Tanzania

**Variable**	**N = 2654**	**% or range**
**Age of the women (mean, range)**	24.61	14–43

**Years of residence in Moshi (mean, range)**	12.75	0–42

**Marital status**		
Married	1624	61.2%
Cohabiting	790	29.8%
Single/divorced/separated	240	9.0%

**Polygamous relationship**	296	11.2%

**Education**		
None	108	4.1%
Primary education	2275	85.6%
Secondary and above	271	10.3%

**Formally employed**	136	5.1%

**Income per month**		
None	762	28.7%
< 30 USD	1726	65.0%
30 USD and above	166	6.3%

*Obstetric history*		

**Parity (Mean, range)**	1.16	0–9

**Gravidity (Mean, range)**	2.35	1–10

**Prior stillbirth/previous pregnancy**	310/1686	18.4%

**History of infant death/previous pregnancy**	141/1686	8.4%

**Age at first pregnancy (mean, range)**	19.77	11–34

*Sexual behavior characteristics*		

**Age at fist debut (mean, range)**	18.30	9–31

**Report casual partners in past 12 months**	117	4.4%

**Lifetime sexual partners (mean, range)**	1.61	1–7

**Condom use at sexual debut**	240	9.0%

**Report of ever use of condoms**	670	25.2%

**Treatment for STI symptoms in past 12 months**	685	25.8%

The prevalence of laboratory diagnosed RTIs by HIV status is shown in Table 2. Seven percent of the women had HIV infection, 0.9% had active syphilis, 33.6% were HSV-2 positive and 17.5% had IgG antibodies for C *trachomatis*. The prevalence of N *gonorrhoeae *was low (0.5%) while 23.9% of the women had trichomoniasis and/or bacterial vaginosis.

**Table 2:** Prevalence of laboratory confirmed reproductive tract/sexually transmitted infections by HIV status among pregnant women in Moshi, Tanzania

	**N**	**%**	**HIV-positive**	**HIV-negative**
**Serology**				

HIV-1	184/2654	6.9%		

Active syphilis	23/2654	0.9%	3.3%	0.7% ***

HSV-2	427/1271	33.6%	43.2%	32.0% **

*Chlamydia trachomatis*	183/1048	17.5%	30.0%	16.6% **

**Cervical & vaginal STIs/RTIs**				

*Neisseria gonorrhoea*	13/2555	0.5%	1.6%	0.4% *

*Trichomonas vaginalis*	129/2555	5.0%	7.1%	4.9%

Bacterial vaginosis	533/2555	20.9%	37.2%	19.6% ***

*Candida albicans*	292/2555	11.4%	14.2%	11.2%

Any vaginal infection^¶^	754/2555	29.5%	46.4%	28.2% ***

**Clinical STIs**				

Genital ulcer	41/2555	1.6%	4.4%	1.4% *

Genital warts	11/2555	0.4%	1.1%	0.4%

Any curable STI **#**	162/2558	6.3%	11.5%	5.9% **

Any curable STI/RTI ^†^	633/2558	24.7%	43.2%	23.3% ***

^¶ ^Trichomoniasis, bacterial vaginosis or candidiasis.

# Syphilis, gonorrhoea, trichomoniasis.

^† ^Syphilis, gonorrhoea, trichomoniasis, bacterial vaginosis.

* p ≤ 0.05, ** p < 0.01, *** p < 0.001

Except for candidiasis, the prevalence of both viral and bacterial RTIs were higher in HIV-seropositive women than in the HIV-seronegative women, significantly for syphilis (p < 0.001), HSV-2 (p = 0.03), gonorrhoea (p = 0.05), Chlamydia trachomatis Ab (p = 0.004), bacterial vaginosis (p < 0.001) and genital ulcers (p = 0.002), Table 2.

Table 3 shows a comparison of RTI prevalence among pregnant women with published work which was carried out in 1999 at the same clinics as the current study [18]. A significant decline in curable RTIs was observed, especially for syphilis (from 3.4% to 0.9%; p = 0.001), trichomoniasis (from 23.4% to 5.0%; p < 0.001) and bacterial vaginosis (from 31.4% to 20.9%; p = 0.001). No significant decrease was observed in the prevalence of HSV-2 infection.

**Table 3:** Trend by age groups of laboratory confirmed STIs/RTIs among pregnant women in Moshi urban, 1999–2004

**STI/RTI**	**15–24 years**	**25–49 years**	**Total**
**Number of participants**			
1999	78	98	176
2002–04	1467	1187	2654

**HSV-2**			
1999	26.9%	41.8%	35.2%
2002–04	28.9%	38.8%	33.5% ↔

**Syphilis**			
1999	1.3%	5.1%	3.4%
2002–04	0.5%	1.3%	0.9% ↓

**Gonorrhoea**			
1999	1.3%	0.0%	0.6%
2002–04	0.6%	0.4%	0.5% ↔

**Trichomoniasis**			
1999	26.0%	21.4%	23.4%
2002–04	5.4%	4.6%	5.0% ↓

**Bacterial vaginosis**			
1999	26.0%	35.7%	31.4%
2002–04	23.4%	17.7%	20.9% ↓

**Any curable STI/RTI**^**†**^			
1999	41.6%	51.0%	46.9%
2002–04	27.2%	21.7%	24.7% ↓

^† ^Syphilis, gonorrhoea, trichomoniasis, bacterial vaginosis.

↓ significant decrease in prevalence; ↔ no change in prevalence.

## Discussion

In this study viral infections (HSV-2 and HIV) are more common than the curable non-viral STIs like trichomoniasis, syphilis or gonorrhoea [14,16,17]. We also noted a decline of curable genital tract infections among pregnant women between 1999 and 2004. A high prevalence of HSV-2 and decreasing prevalence of curable genital tract infections like syphilis, chlamydia, gonorrhoea, trichomoniasis and bacterial vaginosis has been reported in several sub-Saharan countries among pregnant women [15,22,23], among women in the general population [23], and among women in high risk groups [16,17,23]. The introduction of and scaling up of syndromic approach for management of STIs in most primary health clinics may partly explain the decrease [6,23]. The change in the genital infection spectrum however, highlights the need to strengthen STI surveillance, so as to be able to adjust syndromic management protocols according to the epidemiological situation.

Despite the declining trend, the prevalence of trichomoniasis (5%) and bacterial vaginosis (20.9%) are still high, considering that this was a pregnant population. Untreated, trichomoniasis (TV) has been associated with preterm birth and low birth weight (LBW), while bacterial vaginosis (BV) can cause preterm rupture of membranes, preterm birth, LBW and postpartum sepsis especially in women with a previous history of preterm delivery [2]. Prematurity and low birth weight are among the leading causes of perinatal morbidity and mortality in resource-poor settings [1,2,10]. Studies have also indicated that both BV and TV may increase the risk of HIV acquisition, and BV may have an effect on mother-to-child transmission of HIV [10-12]. Efforts are thus required to treat these vaginal infections in pregnancy. An approach like presumptive treatment (mass treatment on the presumption that the disease might be present) has been associated with significant reduction in the prevalence of these infections, and in the incidence of LBW, early neonatal death and preterm delivery [4].

The active syphilis prevalence of 0.9% was similar to that reported for women in the general population (0.2%) or among bar workers (1.1%) in the same district [14,17]. However it was lower than in pregnant women in the Mwanza (7.7%), Mbeya (4.1%) and Kagera (14.9%) regions in Tanzania, and among pregnant women in Uganda (3.3%), Zimbabwe (3.4%), Mozambique (4.7%) and Cameroon (13%), showing variation within and between countries in Africa [3-6,15,22,24]. Our results however might be an underestimation in the prevalence of syphilis because women with early foetal loss resulting from syphilis would not be represented, since we recruited women in the third trimester [3]. Lumbiganon et al (2002) showed that even with a low background prevalence of 0.9%, women with syphilis had significantly more adverse pregnancy outcomes e.g. LBW and perinatal death [25]. Syphilis screening and treatment in pregnancy is thus cost-effective even at prevalences < 1% and supports the WHO recommendation to perform serological screening on all pregnant women at first visit [1]. With resources being mobilized for expansion of prevention of mother-to-child transmission of HIV programs in most sub-Saharan African countries, this opportunity should be used to expand syphilis screening and treatment at the same time.

The higher prevalence of STIs and bacterial vaginosis among HIV-seropositive women than seronegative women has also been reported among pregnant women in Zimbabwe, Cameroon, Thailand and USA [5,8,24]. HIV-infected people have higher rates of genital infection probably because of shared behavioral risk factors that facilitate transmission of both infections [7], or increased susceptibility to some RTIs like gonorrhoea and GUD, especially with advanced immune suppression [26]. RTIs in HIV-infected women has been associated with more severe adverse reproductive health outcomes than in uninfected women, including pelvic inflammatory disease, high grade cervical intraepithelial lesions, postpartum endometrititis, preterm birth and neonatal death [2,24,25]. Also, the presence of bacterial vaginosis, candidiasis, trichomoniasis, gonorrhoeae, chlamydia and HSV-2 in HIV-infected women, increases HIV genital shedding, thus an increased concentration of HIV in genital secretions [9,10,28,29]. Increased infectiousness increases the risk of both vertical and sexual HIV transmission. In fact, studies have recently shown that bacterial vaginosis, chorioamnionitis, genital ulcers and HSV-2 are associated with increased rates of mother-to-child HIV transmission [8,10,30,31]. Efforts to expand HIV-counseling and testing services to all centers offering antenatal care is vital in order to identify HIV-infected women. Apart from offering antiretroviral therapy (ART) to reduce perinatal HIV transmission, the women should also be offered screening for genital tract infections. Treatment of genital infection significantly lowers genital HIV concentrations [9,10,28,29], thus treatment of maternal genital infections in HIV-infected women during pregnancy may substantially reduce negative reproductive effects on the women themselves, may reduce sexual HIV transmission and play a part in reducing mother-to-child transmission. Efforts to address RTIs in HIV-infected women should not be limited to pregnancy but should also be extended to ART care clinics.

There may be possible limitations in the study regarding some of the laboratory tests used to diagnose RTIs. Culture and microscopy were used to diagnose gonorrhoeae and trichomoniasis in this study. Nucleic acid amplification technology using polymerase chain reaction (PCR) has been shown to have a higher sensitivity and specificity than culture and microscopy for identification of both gonorrhoea and trichomoniasis [32,33]. The traditional tests used might have thus missed a substantial proportion of these infections. Further, we used an IgG antibody test to diagnose Chlamydia trachomatis. This test can only show the proportion of women who had been exposed to the Chlamydia infection in the past. It cannot differentiate between women with active and with past infections. Therefore it may be used only for epidemiological studies and not for diagnosing women for treatment.

Despite the possible limitations, the study has demonstrated that STIs and other reproductive tract infections are still prevalent among pregnant women in the area. Routine screening and treatment during antenatal care is recommended. HIV-infected women should receive adequate screening for genital tract infections during pregnancy. Future research and public health preventive efforts should target not only the classical bacterial RTIs but also HSV-2 as it was the most prevalent STI. Lastly, studies using more sensitive assays (PCR) for screening STIs during pregnancy are required, in order to give a more precise picture of STI occurrence.

## Competing interests

The authors declare that they have no competing interests.

## Authors' contributions

SEM designed the study, recruitment of participants, collected and entered data, analyzed data and drafted the manuscript. JU participated in data collection, laboratory testing and data analysis, revising the drafted manuscript. AH conception, design, analysis and reviewed the drafted manuscript. EM designed the study, participated in analysis and interpretation, reviewed the drafted manuscript. SJ design of laboratory testing, chlamydia testing, interpretation of results. NES designed the study, supervised laboratory testing, reviewed the drafted manuscript. BSP conception, design, coordinated the study, interpreted data and reviewed the drafted manuscript. All authors read and approved the manuscript.
